# First postoperative day review after uneventful phacoemulsification cataract surgery: Is it necessary?

**DOI:** 10.1186/1756-0500-5-333

**Published:** 2012-06-27

**Authors:** Irini P Chatziralli, Theodoros N Sergentanis, Evgenia Kanonidou, Leonidas Papazisis

**Affiliations:** 1 Department of Ophthalmology, General Hospital of Veroia, Veroia, Greece; 2 Department of Epidemiology and Biostatistics, Medical School, National University of Athens, Athens, Greece; 3 28, Papanastasiou street, Agios Dimitrios,, 17342, Athens, Greece

**Keywords:** Cataract, Review, Postoperative, First day

## Abstract

**Background:**

Our purpose was to examine the value of the first postoperative day review after uneventful phacoemulsification cataract surgery.

**Methods:**

291 patients who underwent uneventful phacoemulsification were randomized into two groups (ClinicalTrials.gov Identifier: NCT01247155): i) Next day review (NDR group, n = 146) and ii) No next day review (NNDR group, n = 145). The rate of complications, percentage of patients seeking non-scheduled medical consultation up to postoperative day 14, presence of any inflammation-related sign and best corrected visual acuity (BCVA) on postoperative day 28 were analyzed.

**Results:**

In the NDR group, 5.5% of patients developed a postoperative complication, whereas the respective rate was 6.2% in the NNDR group. The difference was not statistically significant (p = 0.791). The most frequent complications were: elevated intraocular pressure, allergy to postoperative treatment, corneal abrasion, punctuate epitheliopathy, iris prolapse and postoperative hyphema, whose rates did not differ between the two groups. The rate of patients seeking non-scheduled medical consultation up to postoperative day 14, presence of any inflammation-related sign, as well as BCVA on day 28 did not exhibit any significant differences between the study groups.

**Conclusions:**

First postoperative day review could be omitted in cases of uneventful cataract surgery.

## Background

Cataract surgery is one of the most common surgical procedures worldwide [1,2]. Phacoemulsification and recent advances in cataract surgery techniques lead to improved postoperative outcomes, as well as to reduced intra- and postoperative complications [2,3]. Therefore, there is a tendency in discharging patients on the same day of surgery, without first day postoperative review [3-6], which is considered to be an increasing issue due to economic reasons. Nevertheless, routine review on the first day postoperatively has several advantages, such as the early detection of complications, reassurance for the patient and training of staff [3]. As a result, there have been studies which stated that first day postoperative review after uneventful phacoemulcification cataract surgery is necessary and others that could not make specific recommendations for the necessity of it [7-9].

Under the light of the above, the purpose of this randomized trial was to examine the value of the review on the first-postoperative day after uneventful phacoemulsification cataract surgery. Three outcomes were adopted in this study, so as to yield a global, comprehensive approach: i. percentage of patients seeking non-scheduled medical consultation up to postoperative day 14, ii. presence of any inflammation-related signs (corneal edema, Tyndall effect, conjunctival hyperemia) [10] on postoperative day 28 and iii. best corrected visual acuity (BCVA) on postoperative day 28.

## Methods

The patients were recruited from the Department of Ophthalmology, General Hospital of Veroia, Veroia, Greece over a 10-month period. Patients were randomly selected from the grand pool of phacoemulsification procedures taking place in the Department; the random selection was based on random numbers allocation, so as to eliminate any selection bias. 311 patients were asked to participate and 304 consented (participation rate: 97.7%). Patients’ recorded data included age, sex, current smoking habits and clinical features, such as presence of hypertension, diabetes mellitus (with or without diabetic retinopathy), pseudoexfoliation, glaucoma and age-related macular degeneration. All patients underwent a full ophthalmologic examination preoperatively i.e., measurement of BCVA (Snellen chart), slit lamp examination, tonometry and fundoscopy in addition to a complete medical history. The study was in accordance with the Declaration of Helsinki and has been approved by the Institutional Review Board of General Hospital of Veroia, Veroia, Greece. Written informed consent was obtained from all patients.

All patients underwent routine phacoemulsification cataract surgery with posterior chamber intraocular lens implantation by the same consultant surgeon (LP) and were randomized to one of the two postoperative follow-up groups: i) Next day review (NDR group, n = 146) and ii) No next day review (NNDR group, n = 145). Patients assigned to the NDR group stayed at the hospital for the first postoperative night, while a doctor examined them in the following morning (slit lamp examination, tonometry). On the other hand, patients allocated to the NNDR group were discharged 3–4 hours after surgery.

Exclusion criteria were the following: i) intra-operative complications, such as posterior capsule rupture, vitreous loss, lost nucleus, zonule dehiscence and wound leak, ii) inadequate social support for overnight care at home, iii) severely limited visual potential in the fellow eye, iv) uveitis or ocular trauma, v) severe systemic diseases limiting activity, vi) patients with learning disability or dementia.

All patients received the same postoperative treatment i.e., combination of tobramycin 0.3% - dexamethasone 0.1% (TobraDex®, Alcon), one drop four times/day, plus ketorolac tromethamine 0.5% (Acular®, Allergan), one drop three times/day. The topical treatment was administered for 28 days after phacoemulsification. Appropriate postoperative instructions were given to all patients in addition to a contact telephone number for emergencies. Two follow-up visits were scheduled for all patients: one on postoperative day 14 and one on postoperative day 28. On postoperative day 14 slit lamp examination, tonometry and fundoscopy were performed; special attention was paid to record whether the patient had sought non-scheduled medical consultation up to postoperative day 14. On postoperative day 28 slit lamp examination, tonometry, fundoscopy and BCVA measurement were conducted. All patients were evaluated by the same team having performed the phacoemulsification procedures and specifically by two independent examiners.

Three outcomes were adopted in this study: i. percentage of patients seeking non-scheduled medical consultation up to postoperative day 14, ii. presence of any inflammation-related signs (corneal edema, Tyndall effect, conjunctival hyperemia) on postoperative day 28 and iii. BCVA on postoperative day 28.

The differences in baseline characteristics, incidence of postoperative complications between the two groups, as well as between outcomes were compared by Chi-square test or Fisher’s exact test (concerning categorical variables) or Mann–Whitney-Wilcoxon (MWW) test for independent samples (concerning continuous variables), as appropriate. Concerning BCVA, the descriptive statistics of the log of the minimum angle of resolution (logMAR) were computed as appropriate [11]. Statistical analysis was performed with STATA 8.0 statistical software (StataCorp, College Station, TX, USA).

## Results

**Figure 1:**
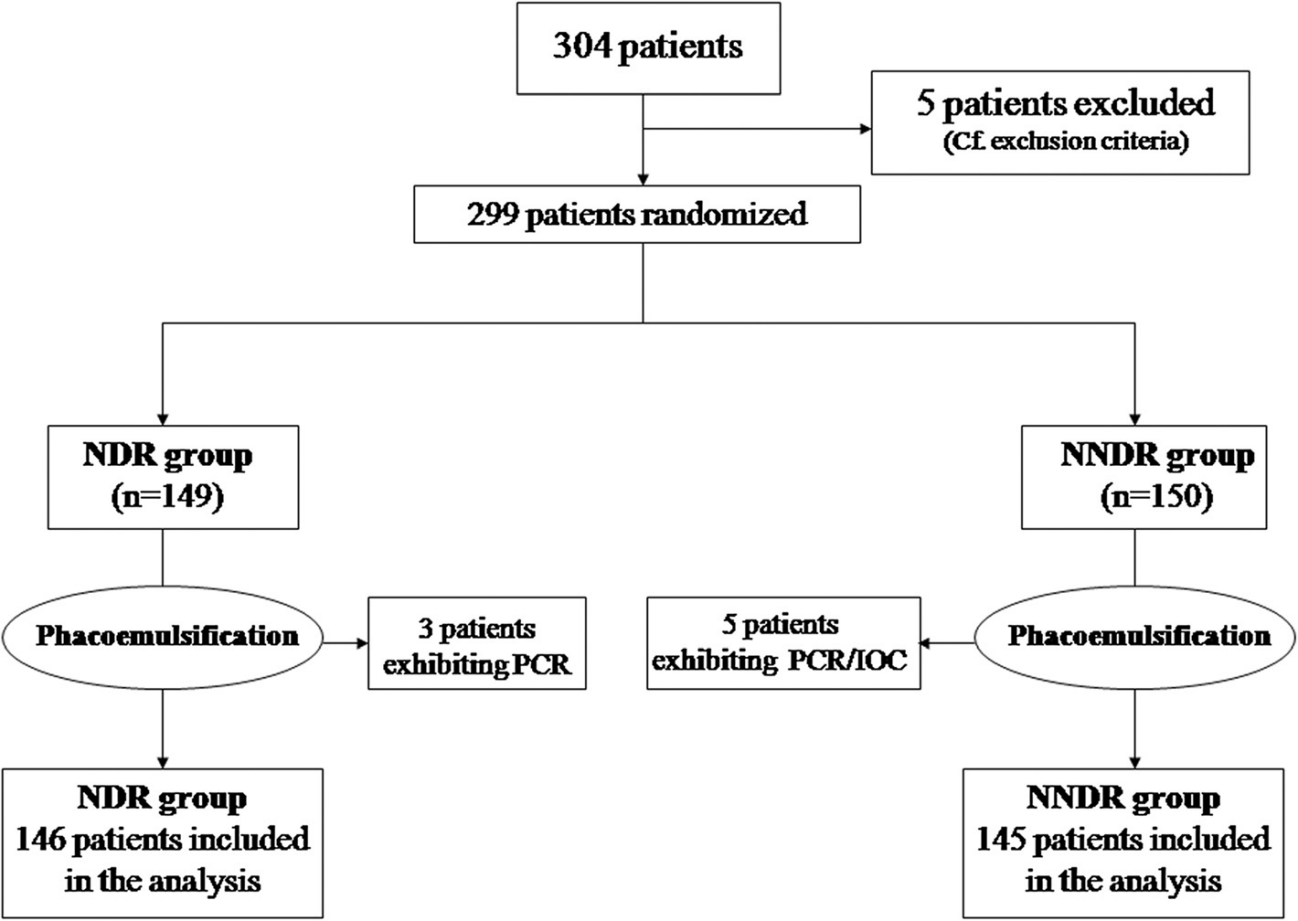
**Flow chart depicting the exclusion criteria and randomization of patients.** NDR: next day review; NNDR: non next day review; PCR: posterior capsule rupture; IOC: intra-operative complications.

The study design, as well as the randomization of patients in the two groups, is depicted in the respective flow chart (Figure 1). Table 1 represents the baseline demographic, clinical features and lifestyle habits of the study groups. The postoperative complications with their statistical significance are illustrated in Table 2. Of the 146 patients randomized to NDR group, eight (5.5%; 95%CI: 2.4%-10.5%) developed a postoperative complication vs. nine (6.2%; 95%CI: 2.9%-11.5%) of 145 patients in the NNDR group. The difference was not statistically significant (p = 0.791, Chi-square test). Two patients (one in the NDR group and one in the NNDR group) presented with coexistent corneal abrasion and punctuate epitheliopathy; as a result, the total number of complications in the NDR and NNDR group were nine and ten, respectively.

**Table 1:** The baseline features of the two study groups

**Continuous variables**	**NDR group** **(n = 146)**	**NNDR group** **(n = 145)**	**p**
	**mean ± SD**	**mean ± SD**	
Age (years)	75.4 ± 7.2	75.8 ± 7.0	0.584^MWW^
BCVA prior to phacoemulsification (logMAR)	0.59 ± 0.13	0.63 ± 0.14	0.193^MWW^
**Categorical and ordinal variables**	**N (%)**	**N (%)**	
Sex (male)	73 (50.0)	79 (54.5)	0.444^C^
Current smoking (yes)	24 (16.4)	20 (13.8)	0.529^C^
Hypertension (yes)	104 (71.2)	113 (77.9)	0.190^C^
Diabetes mellitus (yes)	24 (16.4)	23 (15.9)	0.894^C^
Diabetic retinopathy (yes)	14 (9.6)	11 (7.5)	0.530^C^
Glaucoma (yes)	13 (8.9)	17 (11.7)	0.429^C^
Pseudoexfoliation (yes)	44 (30.1)	35 (24.1)	0.250^C^
Age-related macular degeneration (yes)	11 (7.5)	10 (6.9)	0.834^C^

^MWW^p-value derived from Mann–Whitney-Wilcoxon test for independent samples, ^C^p-value derived from Pearson’s chi-square, ^F^p-value derived from Fisher’s exact test.

**Table 2:** The profile of complications in the two study groups

**Complications**	**NDR group**	**NNDR group**	p*
	**N(%)**	**N(%)**	
*Postoperative complications*			
Allergy to postoperative treatment	3 (2.1)	1 (0.7)	0.622
Iris prolapse	0 (0.0)	1 (0.7)	0.498
Corneal abrasion	2 (1.4)	2 (1.4)	>0.999
Elevated intraocular pressure (>30 mmHg)	2 (1.4)	4 (2.8)	0.447
Punctuate epitheliopathy	2 (1.4)	1 (0.7)	>0.999
Postoperative hyphema	0 (0.0)	1 (0.7)	0.498

*p-values derived from Fisher’s exact test.

Worthy of note, regarding NDR group, all complications appearing in Table 2 became evident on next-day review, except for allergy to postoperative treatment which prompted patients to seek non-scheduled medical consultation (see below). With respect to NNDR group, all complications had become evident prior to postoperative day 14, either at the scheduled visit or at a non-scheduled consultation (two patients, see below).

Concerning the three outcomes of the study, no statistically significant differences were noted (Table 3). The percentage of patients seeking non-scheduled medical consultation up to postoperative day 14 was minimal in both groups; accordingly, the frequency of any inflammation-related sign was scarce on postoperative day 28. It is worth mentioning that the underlying cause in all three patients seeking non-scheduled medical consultation up to postoperative day 14 in NDR group was allergy to postoperative treatment (allergy emerged on postoperative day 2 for two patients and on postoperative day 3 for one patient); on the contrary, the underlying cause for the respective two cases in NNDR group was corneal abrasion (the two patients sought non-scheduled medical consultation due to pain on postoperative day 5 and 9, respectively). BCVA on postoperative day 28 did not exhibit statistically significant difference between the two groups (0.06 ± 0.08 vs. 0.06 ± 0.06 for NDR and NNDR respectively, p = 0.859, MWW). Of note, the agreement between the two examiners was 100%, regarding the assessment at day 14 and day 28.

**Table 3:** Frequency of the study outcomes in the two study groups

**Categorical outcomes**	**NDR group**	**NNDR group**	**p**
	**N(%)**		
Seeking non-scheduled medical consultation up to postoperative day 14	3 (2.1)	2 (1.4)	>0.999^F^
Presence of any inflammation-related sign on postoperative day 28	2 (1.4)	2 (1.4)	>0.999^F^
**Continuous outcome**	**mean ± SD**		
BCVA on postoperative day 28	8.77 ± 1.27	8.85 ± 1.13	0.859^MWW^

^F^p-value derived from Fisher’s exact test, ^MWW^p-value derived from Mann–Whitney-Wilcoxon test for independent samples.

## Discussion

The principal message of this study is that the first postoperative day review could be omitted after uneventful phacoemulsification cataract surgery. The frequency of serious complications detected on the first postoperative day was low and there was no statistically significant difference between the two groups concerning non-scheduled consultation, inflammation-related signs or postoperative complications. Moreover, BCVA seems not to be affected by the first day postoperative review, as Tinley et al. have also noted [4].

Regarding complications, iris prolapse is rare after small incision cataract surgery and is thought to be associated with poor wound construction or postoperative manipulation [8]. Allergy to postoperative treatment is not a vision-threatening complication and can be predicted based on a thorough medical history [12]. With respect to corneal abrasion, pain was the symptom that led patients to seek non-scheduled advice, when first postoperative day review had been withdrawn. Pain may be indeed an alarming sign for punctuate epitheliopathy as well [13]. Worthy of note, the meaningful triad of inflammation-related signs i.e., corneal edema, Tyndall effect, conjunctival hyperemia seemed to follow the same trend in both groups [10].

Overall, the most frequent postoperative complication in our study was elevated intraocular pressure. This is in agreement with the findings of previous investigations [3,7,8]. Interestingly, Dinakaran et al. highlighted that first postoperative day review is necessary, so as to check intraocular pressure [7]. However, it has been shown that the postoperative rise in intraocular pressure is transient as its peak occurs between 3 and 6 hours postoperatively [14]. Of note, intraocular pressure elevation is more common in the subset of patients with coexisting glaucoma and can be prevented by using prophylactic topical intraocular pressure lowering agents [3,15,16]. Taken as whole, first postoperative day review may offer little to the reduction of intraocular pressure.

The rationale for examining patients on the first postoperative day pertains to detect treatable early complications. Furthermore, patients feel more reassured and can be educated in postoperative care and drop installation [3,8]. Nevertheless, severe complications, such as endophthalmitis and retinal detachment, are rare and not necessarily detected on the first postoperative day [3,4,8], as our study also suggests. As a result, it seems better to have a review on postoperative day 3 to 4 instead of first postoperative day review. It is also important to ensure that all patients might easily have access to eye care providers postoperatively at any time, so as to be appropriately advised if they have any discomfort or sight-threatening symptom.

A meaningful limitation of this study that should be declared pertains to the design of the study. A blind (masked) assessment at day 14 and 28 was not feasible, as the team of surgeons performing the phacoemulsification procedure were essentially the same as the evaluation team. Nevertheless, it should be stressed that two independent examiners evaluated the patients with 100% agreement, a fact pointing to the rather negligible effect mediated by the non-blind evaluation.

## Conclusion

In conclusion, this study indicates that first postoperative day review could be omitted in cases of uneventful phacoemulsification cataract surgery, supplemented by patients-initiated review in the interim.

## Abbreviations

BCVA, Best corrected visual acuity; NDR, Next day review; NNDR, No next day review; MWW, Mann–Whitney-Wilcoxon.

## Authors’ contributions

IC conceived the idea of the study, designed the study, collected data and drafted the manuscript. TS performed the statistical analysis and drafted the manuscript. EK collected data and revised critically the manuscript. LP conceived the idea of the study and revised critically the manuscript. All authors read and approve the final manuscript.

## Competing interests

The authors declare that they have no competing interests.
